# Adaptive Disconnector States Diagnosis Method Based on Adjusted Relative Position Matrix and Convolutional Neural Networks

**DOI:** 10.3390/s25061701

**Published:** 2025-03-10

**Authors:** Peifeng Yan, Chenzhang Chang, Dong Hua, Haomin Huang, Suisheng Liu, Peiyi Cui

**Affiliations:** 1 School of Electric Power Engineering, South China University of Technology, 381 Wushan Road, Tianhe District, Guangzhou 510641, China; 202221015192@mail.scut.edu.cn (P.Y.); 202321014290@mail.scut.edu.cn (H.H.); 2 School of Science and Engineering, Chinese University of Hong Kong, 2001 Longxiang Avenue, Longgang District, Shenzhen 518172, China; 120090204@link.cuhk.edu.cn; 3 Guangdong KingWa Energy Technology Co., Ltd., No. 88, Industry Avenue North, Guangzhou 510000, China; samson.ss.liu@gmail.com (S.L.); cuipeiy123@126.com (P.C.)

**Keywords:** high voltage disconnector, states diagnosis, relative position matrix, convolutional neural network

## Abstract

Due to long-term outdoor working, High-Voltage Disconnectors (HVDs) are prone to potential faults. Currently, most studies on HVD state diagnosis methods have tested only one type of HVD, and the generalization capability of these methods for other HVDs has not been verified. In this paper, we propose an HVD state diagnosis method featuring adaptive recognition capabilities based on Fault Difference Signals, Adjusted Relative Position Matrix and Convolutional Neural Networks (FDS-ARPM-CNN). First, we align the measured operational power signal of the HVD drive motor with the recorded normal operational power signal, deriving the FDS through subtraction. Next, to address the issue of traditional Relative Position Matrix (RPM) conversion processes that lose sample amplitude information, we introduce a targeted improvement to the relative position matrix calculation method, converting the one-dimensional FDS into a two-dimensional image. Finally, we achieve high-accuracy diagnosis and classification of HVD states using a CNN that incorporates Batch Normalization (BN) and GELU activation functions. Experimental validation demonstrates that the neural network model, trained on one model of HVD, maintains strong generalization capabilities on data from other HVD models. This method effectively alleviates the challenges of acquiring fault samples in data-driven approaches for HVD state diagnosis, showcasing significant practical value.

## 1. Introduction

Fault diagnosis of power equipment has been a prominent topic in the field of intelligent operation and maintenance of power generation. High-Voltage Disconnectors (HVDs) are among the most numerous primary equipment used in substations, playing a crucial role in power system operation scheduling and substation maintenance [1,2,3]. However, due to long-term exposure of their mechanical components to outdoor atmospheric conditions, high-voltage disconnectors are prone to mechanical failures such as component corrosion, mechanism deformation, and lack of lubrication in moving joints. These issues can lead to abnormal closing and opening operations. Therefore, it is essential to conduct an in-depth study on the state diagnosis of HVDs [4,5].

The most widely accepted intelligent diagnostic method for HVDs is based on computer vision technology to autonomously identify the open and closed states of HVDs [6,7,8]. However, many studies have not validated whether this method can still accurately recognize the closed state in cases of incomplete closing. Furthermore, this method is significantly affected by the shooting angle and potential obstructions.

Recent studies by researchers have focused on the other state characteristics of HVDs. When equipment such as the Gas Insulated Switchgear (GIS) and HVD is operated, vibration signals are generated due to the interaction of their mechanisms [9]. With changes in equipment states, the frequency and amplitude of the vibration signals will vary accordingly. Techniques like empirical mode decomposition [10], variational mode decomposition [11] and empirical wavelet transform [12] are utilized to extract valid information from the vibration signals generated by GIS. In response to the limitations of traditional feature extraction methods, which are often too direct and insufficient in extracting feature information [13], segments the signal and reconstructs the phase space for signals at different stages, thereby enhancing local feature information. Reference [14] employs a generalized S-transform matrix to extract the original time-frequency characteristics and uses the gray-level co-occurrence matrix to extract texture features from the signal, allowing for a balanced consideration of both global and local information. However, the collection of vibration signals will be affected by the installation accuracy of sensors.

In [15], the rotation angle of the HVD was measured by installing a magnetic-sensing angle transducer on the rotating spindle, and the rotation angle-time signals were plotted. When an additional clamping stagnation force was applied to the operating mechanism, the rotation angle-time signal distorted. However, for HVDs with small and uniform operating torque, their sensitivity to certain faults is insufficient. When the HVD is operated, the motor shaft controls the spindle to drive the entire action mechanism, so the control torque of the spindle can directly reflect the mechanism state of the HVD. Reference [16] proposed a hand-swing torque detection method to measure the operating torque based on wireless transmission and determine the states using an expert system. However, the detected data fluctuated significantly due to variations in manual operations. The author of [17] directly measured the operating torque and spindle rotation angle by installing dynamic torque sensors and encoders in the operating holes of the HVD operating mechanism box. By plotting the torque-angle signals and comparing the average values, extreme values, and differences at the same rotation angles, the state of the HVD could be identified, and the probable position of the mechanism fault could be located. Reference [18] collected motor current signals using closed Hall current sensors when the HVD was activated and employed the Hilbert transform to extract the envelope of the current signal. Experiments demonstrated that changes in motor torque correspond to changes in stator current, making it a useful characteristic for HVD diagnosis.

To further improve the recognition accuracy of the HVD state, researchers have considered the recognition effect of different features on various faults and have combined multiple feature signals [19,20]. These studies combine the advantages of detection sensitivity from both vibration signals and motor current by fusing these two types of feature information, resulting in high recognition accuracy during the diagnosis and classification phase. However, these studies require complex feature extraction processes and suffer from the drawback of insufficient applicability of the feature extraction methods. Additionally, the use of motor current detection does not take into account the changes in power factors during the operation of the motor.

The methods mentioned above are only applicable for measuring the characteristic signals of a single HVD. Achieving large-scale sensor installation and transformation for HVDs already deployed in substations involves a significant workload. Meanwhile, the aforementioned studies do not compare the differences in signals from different HVD devices or validate the applicability of the methods.

With the continuous development of computer technology, data-driven algorithms based on deep learning and machine learning have been widely applied in the field of fault diagnosis [21]. These models have garnered significant attention due to their high diagnostic accuracy and convenience. By leveraging existing data and labels, the models can mine and learn the mapping relationship between features and labels in the data, allowing them to make autonomous decisions. References [22,23] developed a Back Propagation (BP) neural network and introduced a Particle Swarm Optimization (PSO) technique to avoid local optima in the BP algorithm, achieving high-precision circuit breaker fault diagnosis. Reference [24] utilized Support Vector Machines (SVM) and employed Kernel Principal Component Analysis (KPCA) to reduce the dimensionality of the input data through principal component extraction from the original features, enabling efficient fault diagnosis of high-voltage disconnectors within Gas Insulated Switchgear (GIS) with a limited number of samples. Reference [25] constructed a network model for bearing fault diagnosis based on Bidirectional Long Short-Term Memory (Bi-LSTM). The model leverages the ability of LSTM to effectively capture and retain long-term dependency information, combined with transfer learning strategies to achieve fault recognition in bearings with limited samples. Reference [26] applied transformer neural networks to the fault diagnosis of rotating components, utilizing the attention mechanism within the model to enhance critical features, resulting in superior recognition accuracy.

Data-driven models rely on a sufficient number of data samples. However, in reality, there are many different types of HVDs, each often having distinct operating mechanisms, resulting in varying operational characteristic data. Moreover, HVDs can exhibit multiple abnormal states. It is also challenging to obtain sample data for these abnormal states. Therefore, for each state, if each type of HVD requires multiple samples to support the initial training of models, it would significantly hinder the practical application of these methods.

Based on the above analysis, we proposed an adaptive HVD state diagnosis method. Voltage and current data are collected by installing a sensor at the power supply branch of the HVD motor in the substation, and the power signal is then calculated. Leveraging the similarity in the effects of the same type of fault on HVD operation, the measured action signals after filtering are aligned with the normal action signals, and the differences are calculated to obtain the Fault Difference Signals (FDS). An Adjusted Relative Position Matrix (ARPM) matrix transformation is then introduced to convert the time-series signals into two-dimensional images, thereby enhancing the relative information of the time-series data points. Finally, an improved AlexNet (IAlexNet) Convolutional Neural Network (CNN) is utilized to diagnose the states, achieving high-accuracy state recognition for trained HVDs, while also demonstrating good generalization capabilities for untrained HVDs. This approach effectively alleviates the challenges posed by the diversity of HVD types and the difficulty in collecting fault samples, while also reducing the difficulty and cost of application.

The structure of this paper is organized as follows: The methodology is presented in Section 2. Section 3 introduces the experimental pre-work. Section 4 presents the verification and analysis of the results obtained. Section 5 offers a comprehensive summary.

## 2. Materials and Methods

**Figure 1:**
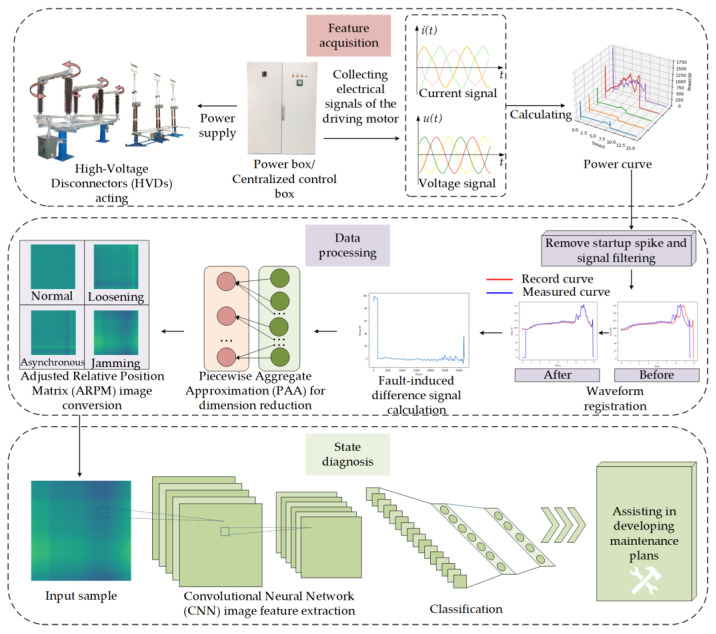
The framework of the proposed method.

The framework of the adaptive HVD recognition method based on FDS-ARPM-CNN proposed in this paper is shown in Figure 1. The workflow of this method consists of four main parts:

(1) Feature acquisition stage: By installing sensors in the control cabinet or centralized power supply cabinet, the data of a single operation of multiple HVDs in the substation can be measured, and the operational power signal can be calculated.
(2) Data processing stage: First, the starting peak of the newly measured power signal is eliminated, and the signal is filtered, and then it is aligned with the recorded normal working state signal of the HVD to eliminate the influence of the inconsistent mechanical stroke caused by the maintenance or debugging of the HVD. Then, the power signal is subtracted from the Normal State (NS) signal of the HVD to obtain the possible fault-induced difference signal, called the FDS. The FDS is subjected to Piecewise Aggregate Approximation (PAA)for dimensionality reduction and correlation processing to obtain the relative position matrix, which is subsequently converted into a heat map.
(3) State diagnosis stage: First, the CNN is used to extract features from sample ARPM images and learn fault characteristics. Finally, the state diagnosis result is obtained through the classifier, which can assist substation operation and maintenance personnel in formulating a reasonable maintenance plan.

The following content will provide a detailed explanation of the data processing methods and the design of the state diagnosis model.

### 2.1. Waveform Registration

Cross-correlation can be used for aligning two signals by measuring their similarity at different time shifts or lags [27]. It works by shifting one signal relative to the other and computing their pointwise product, summed over all time points, to determine the degree of overlap at each shift. The lag at which the cross-correlation reaches its maximum value indicates the optimal alignment between the two signals.

Given two discrete time signals X={x[i],i∈[0,N)} and {Y=y[j],j∈[0,M)}, the cross-correlation $R_{xy}[m]$ between the X and Y is given by:

(1) $$R_{xy}[m]=\left\{\begin{array}{ll} \sum_{n=m}^{N-1}\hat{x}[n]\cdot \hat{y}[n-m], & \mathrm{for}\;0\leq m<N-1\\ \sum_{n=0}^{M+N-m-2}\hat{x}[n]\cdot \hat{y}[n+m-N+1], & \mathrm{for}\;N-1\leq m<M+N-1 \end{array}\right.,$$

where $\hat{x}[n]=\left\{\begin{array}{ll} x[i], & \mathrm{for}\;0\leq i<N\\ 0, & \mathrm{others} \end{array}\right.$ and $\hat{y}[n]=\left\{\begin{array}{ll} y[j], & \mathrm{for}\;0\leq j<M\\ 0, & \mathrm{others} \end{array}\right.$.

However, due to the susceptibility to significant changes in local signals, the alignment performance of this method is not ideal.

This paper presents an improved signal alignment method based on cross-correlation, utilizing signal fluctuation characteristic points for registration between two signals. The algorithm first calculates the gradients of both signals and quantizes these gradients. Then, it computes the cross-correlation between the two gradient signals. Subsequently, based on the cross-correlation coefficient, the signals undergo shift and alignment processing. When signals of unequal lengths emerge after alignment, zero-padding is applied either at the beginning or end of the signals to ensure the measured signal and the normal operational signal are of equal length.

Algorithm 1 describes the steps of aligning and registering the measured operational signals with the recorded NS operational signals. Subsequently, based on the registered signals, the two signals are subtracted to obtain the FDS.
**Algorithm 1:** Waveform registration and FDS calculating
**Input**: the discrete signal under NS **SignalA**, the new measured signal **SignalB** **Output**: **FDS**1:**GradA** ← Difference(**SignalA**), **GradB** ← Difference(**SignalB**)//Calculate gradients
//Step 1. Quantize gradients to {−1, 0, 1}2:i ← 03:**while** (i < length(**GradA**)) **do**4:    **if** (**GradA**[i] > 0) **then**5:        **GradA**[i] ← 16:    **else if** (**GradA**[i] < 0) **then**7:        **GradA**[i] ← −18:    **end if**9:    i ← i + 110:**…** //Repeat quantization for GradB
//Step 2. Calculate cross-correlation and find the aligned point11:**xcorr** ← CrossCorrelate(**GradA**, **GradB**) //Cross-correlation calculating12:maxIndex ← ArgMax(**xcorr**) //Find the index value of the maximum value13:lag ← maxIndex − length(**SignalB**) + 1 //find the aligned point
// Step 3. Align both signals and use zero padding to equal both signals14:**if** (lag < 0) **then**15:    **SignalA** ← ZeroPad(**SignalA**, -lag, “front”) //Parameter -> (source signal, padding number, padding direction)16:**else if** (lag > 0) **then**17:    **SignalB** ← ZeroPad(**SignalB**, lag, “front”)18:**end if**19:**if** (length(**SignalB**) > length(**SignalA**)) **then**20:    diffLength ← length(**SignalB**) − length(**SignalA**)21:    **SignalA** ← ZeroPad(**SignalA**, diffLength, “end”)22:**else if** (length(**SignalA**) > length(**SignalB**)) **then**23:    diffLength ← length(**SignalA**) − length(**SignalB**)24:    **SignalB** ← ZeroPad(**SignalB**, diffLength, “end”)25:**end if**26:**return FDS** ← **SignalA** − **SignalB**

### 2.2. Relative Position Matrix

The extraction of discriminative features from one-dimensional time series data remains a formidable challenge in pattern recognition algorithms, particularly as conventional neural networks often demonstrate limitations in capturing intricate correlational information within sequential data. In the fields of signal processing and machine learning, a promising approach involves the transformation of one-dimensional temporal sequences into two-dimensional matrix and image representations. This dimensional transformation paradigm offers several benefits. The resultant image representations serve to amplify both the local temporal characteristics and global trend features inherent in the time series data.

Notably, the Relative Position Matrix (RPM)transformation method generates representations that optimize the feature space distribution, characterized by high intra-class cohesion and pronounced inter-class separation [28]. This advantageous property substantially enhances the capacity of subsequent deep learning classification models to establish feature-category mappings, thereby improving overall classification performance.

However, the traditional RPM calculation involves normalizing the signal using Z-score normalization based on the data distribution of each individual sample. This process can result in the loss of the original amplitude information of the signal. Considering that the method used in this paper focuses on converting the fault difference signals of HVD into an RPM, if Z-score normalization is applied, the temporal trend of the signal will be confined to the single sample itself. For example, a signal captured by the sensor under NS may have slight differences from the recorded normal signal, even though they are not exactly the same. When these two signals are subtracted to obtain a fault difference signal, the amplitude of the resulting difference is quite small. After normalization, these small fluctuations are amplified, meaning irrelevant features may be exaggerated, which is detrimental to the learning process of the subsequent classification model.

According to the characteristics of HVD FDS, this paper proposes an adjustment for the RPM conversion process. The Z-score normalization is removed, and the final Min-Max normalization process is adjusted in this paper. The steps for calculating the ARPM are as follows:

1. For time series data $X=[x_{1},x_{2},\ldots ,x_{n}]$ of length *n,* Piecewise Aggregate Approximation (PAA) algorithm reduces the dimensionality of *X* to a new data $\bar{x}={\bar{x}}_{1},{\bar{x}}_{2},\ldots ,{\bar{x}}_{m}$ of length m, where *m* < *n*. The reduction ratio is defined as $c=\left\lfloor n/m\right\rfloor$, which determines the number of data points in each segment. The PAA algorithm can be formally defined as follows:

(2) $${\bar{x}}_{i}=\left\{\begin{array}{ll} \frac{1}{c}\sum_{j=c\times (i-1)+1}^{c\times i}x_{j}, & i=1,2,\cdots ,m-1\\ \frac{1}{n-c\times (m-1)}\sum_{j=c\times (i-1)+1}^{n}x_{j}, & i=m \end{array}\right..$$

This formula computes the mean of the data points in the *i*-th segment, providing a representative value for that segment. The PAA algorithm not only enables dimensionality reduction but also transforms data of varying lengths into a consistent length, making it particularly suitable for different types of HVD operational signals.

2. Calculate the difference between each position in the standardized time series to construct a relative position matrix *M*:

(3) $$\left.M=\left[\begin{array}{cccc} {\bar{x}}_{1}-{\bar{x}}_{1} & {\bar{x}}_{2}-{\bar{x}}_{1} & \cdots & {\bar{x}}_{m}-{\bar{x}}_{1}\\ {\bar{x}}_{1}-{\bar{x}}_{2} & {\bar{x}}_{2}-{\bar{x}}_{2} & \cdots & {\bar{x}}_{m}-{\bar{x}}_{2}\\ \vdots & \ddots & \ddots & \vdots \\ {\bar{x}}_{1}-{\bar{x}}_{m} & {\bar{x}}_{2}-{\bar{x}}_{m} & \cdots & {\bar{x}}_{m}-{\bar{x}}_{m} \end{array}\right.\right].$$

The elements of the relative position matrix link the sequence values at each time point in the original time series. Each row can be viewed as a rearranged time series, using a specific sequence value as a reference point. Meanwhile, each column provides a mirrored perspective of the row, presenting potential features that may be implicitly embedded in the time sequence from a reversed viewpoint.

3. Finally, the adjusted Min-Max normalization process is described as follows:

(4) $$F=\min(\max(\frac{M+\max(X_{\mathrm{Normal}})}{2\times \max(X_{\mathrm{Normal}})},0),1)\times 255,$$

here F represents the adjusted grayscale value matrix, and $X_{\mathrm{Normal}}$ represents the recorded signal under NS.

This method scales the signal using the maximum value of the recorded normal signal, preserving the amplitude characteristics of the fault difference signals within the same type of HVDs. In general, the amplitude of the fault difference signal does not exceed the maximum value of the normal operational signal. Even if the amplitude does exceed this value, capping it at the maximum of the recorded normal signal does not affect the integrity of the fault characteristics.

### 2.3. CNN Structure

AlexNet is a landmark convolutional neural network architecture that significantly advanced the field of deep learning in computer vision. It utilized a relatively deep architecture with five convolutional layers followed by three fully connected layers, incorporating ReLU activations for faster training, max pooling for dimensionality reduction, and dropout for regularization to combat overfitting. AlexNet can automatically extract features from images, reducing the need for manual feature engineering. Due to its simple and effective network architecture as well as fast and efficient training capabilities, AlexNet still has a wide range of applications in simple image classification tasks [29].

However, AlexNet has its limitations; it has a relatively large number of parameters, making it prone to overfitting, especially on smaller datasets and without proper regularization. Therefore, this paper improves AlexNet and designs a more effective recognition network, as shown in Table 1, tailored for the classification task at hand.

In this study, the optimization work is reflected in the following two perspectives:

1. Add a Batch Normalization (BN) layer after each convolutional layer to achieve regularization and reduce the risk of overfitting in the model.
2. Replacing the activation function ReLU with GELU in the original model avoids the risk of neuron death and enhances the expressive power of the model.

Deep neural networks, such as AlexNet, often suffer from the problem of internal covariate shift, where the distribution of layer inputs changes during training as the parameters of the previous layers are updated. This shifting distribution can slow down the training process and make it difficult to tune hyperparameters effectively, such as learning rates. BN helps to mitigate this problem by normalizing the inputs to each layer, ensuring that they maintain a consistent distribution throughout training [30].

**Figure 2:**
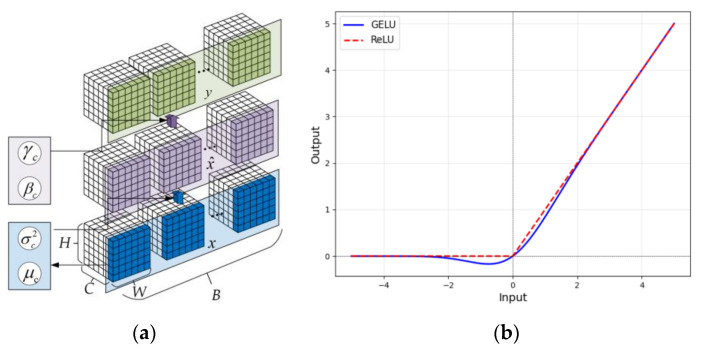
Optimization work. (**a**) Operating process of BN, (**b**) ReLU and GELU activation function.

BN operates on mini-batches of data during training, as shown in Figure 2a, and involves the following steps:

1. For an 2D input x with batch size B, channel number C, height H, and width W, the mean μ and variance $\sigma^{2}$ of channel c are computed as follows:
  (5) $$\mu_{c}=\frac{1}{B\times H\times W}\sum_{b=0}^{B-1}\sum_{i=0}^{H-1}\sum_{j=0}^{W-1}x_{\left(b,c,i,j\right)},$$
  (6) $$\sigma_{c}^{2}=\frac{1}{B\times H\times W}\sum_{b=0}^{B-1}\sum_{i=0}^{H-1}\sum_{j=0}^{W-1}{\left(x_{\left(b,c,i,j\right)}-\mu_{B}\right)}^{2}.$$
2. The input x is normalized using the calculated mean and variance as following:
  (7) $${\hat{x}}_{\left(b,c,i,j\right)}=\frac{x_{\left(b,c,i,j\right)}-\mu_{c}}{\sqrt{\sigma_{c}^{2}+\epsilon }},$$
  here ϵ is a small constant added for numerical stability to prevent division by zero.
3. After normalization, BN introduces learnable parameters γ and β to allow the model to retain the capacity for representation,
  (8) $$y_{\left(b,c,i,j\right)}=\gamma_{c}{\hat{x}}_{\left(b,c,i,j\right)}+\beta_{c},$$
  here $y_{\left(b,c,i,j\right)}$ is the output after batch normalization for the input $x_{\left(b,c,i,j\right)}$.

GELU introduces a degree of uncertainty during training, which can serve as a form of regularization, reducing the risk of overfitting. This implies that models utilizing GELU typically exhibit better generalization performance when confronted with new data. Meanwhile, GELU introduces a smoother nonlinear characteristic in the negative value region. In contrast to ReLU, which simply truncates the output to zero for negative inputs, GELU allows some negative values to pass through, preserving more complex feature information and enhancing the model’s nonlinearity. The schematic diagram of the functions of ReLU and GELU is shown in Figure 2b, and the function of GELU can be expressed as follows:

(9) GELU(x)=x⋅Φ(x)

where Φ(x) is the cumulative distribution function of the standard normal distribution, which can be expressed as:

(10) $$\Phi (x)=\frac{1}{2}\left(1+\mathrm{erf}\left(\frac{x}{\sqrt{2}}\right)\right)$$

where erf(x) is Gaussian error function and is defined as:

(11) $$\mathrm{erf}(x)=\frac{2}{\sqrt{\pi }}\int_{0}^{x}e^{-t^{2}}dt.$$

## 3. Experimental Setup and Data Acquisition

### 3.1. Experimental Platform

**Figure 3:**
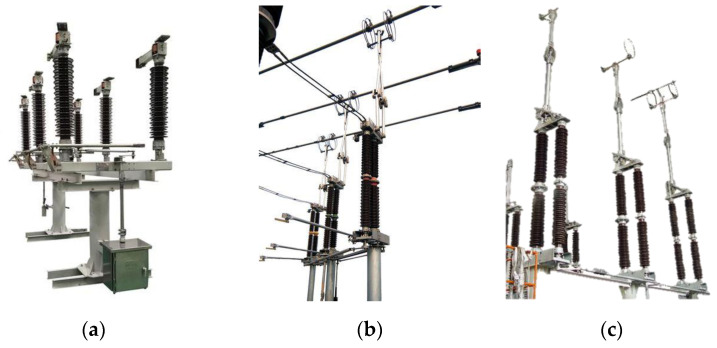
HVDs in experiments. (**a**) GW4-126, (**b**) GW6-252, (**c**) GW22-252.

This study uses the GW4-126, GW6-252 and GW22-252 types of HVDs, as shown in Figure 3. GW4-126 is a double column horizontal rotating structure HVD, where the closing and opening of the contacts are completed by rotation. GW6-252 and GW22-252 are, respectively, single column double arm vertical telescopic structures and single column single arm vertical telescopic HVDs, which achieve circuit opening and closing through vertical separation of contacts. These types of HVDs have been widely adopted in 110 kV and 220 kV substations and transmission lines, making them highly representative of commonly used equipment at these voltage levels.

**Figure 4:**
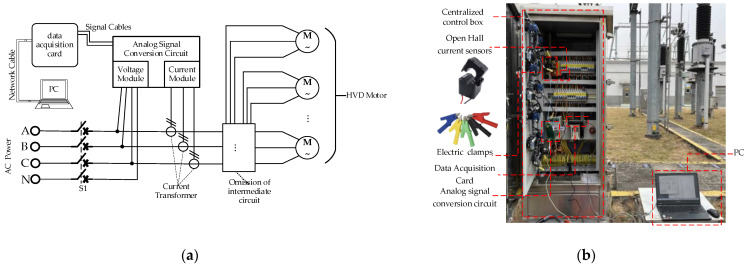
The motor power detection system. (**a**) system wiring diagram, (**b**) on-site wiring diagram.

In this study, the motor power detection system used for data collection consists of open Hall current sensors, electric clamps, an analog signal conversion circuit, a data acquisition card, and a PC, as shown in Figure 4. The parameters of the signal acquisition device are shown in Table 2. During the experiment, the system is installed in a centralized control box that houses multiple HVD motor power supplies.

According to substation electrical equipment operation specifications, during HVD’s operation in a substation, personnel should follow the sequence one by one outlined in the operation ticket, meaning that there is only one HVD operating at a time. Therefore, in practical applications, even if the measured branch supplies power to multiple HVDs, the fact that no two HVDs operate simultaneously within a substation will not affect the measurement of action data and the state diagnosis for an individual HVD.

The installation of the sensor for the detection system does not require any modifications to the original circuit of the centralized control box. For the current sensor, it is only necessary to open the sensor, pass the measured cable through the detection hole, and then close the sensor. For voltage acquisition, the power clamp only needs to be clamped to the AC power breakpoint.

### 3.2. State Simulation Methods

To establish a comparison with NS and to compile a diverse set of working state into a sample library, this study simulated five distinct fault conditions for HVDs, namely: Inadequate Closing (IC), Three-phase Asynchrony (TA), Linkage Decoupling (LD), Light Jamming (LJ) and Severe Jamming (SJ).

During the experiment, in order to prevent damage to the disconnect switch, we developed on-site simulation methods for various mechanical states of the HVD. This was performed under the guidance of substation maintenance experts and based on research materials that identified the causes of different HVD faults. The corresponding simulation methods are detailed in Table 3.

### 3.3. Data Acquisition and Display

Following the simulation of diverse states of the HVD via the aforementioned methodologies, the three-phase voltage and current data were gathered at an acquisition frequency of 4000 Hz. By repetitively engaging and disengaging the disconnector in identical conditions, multiple sets of sample data were amassed to construct the sample library further.

**Figure 5:**
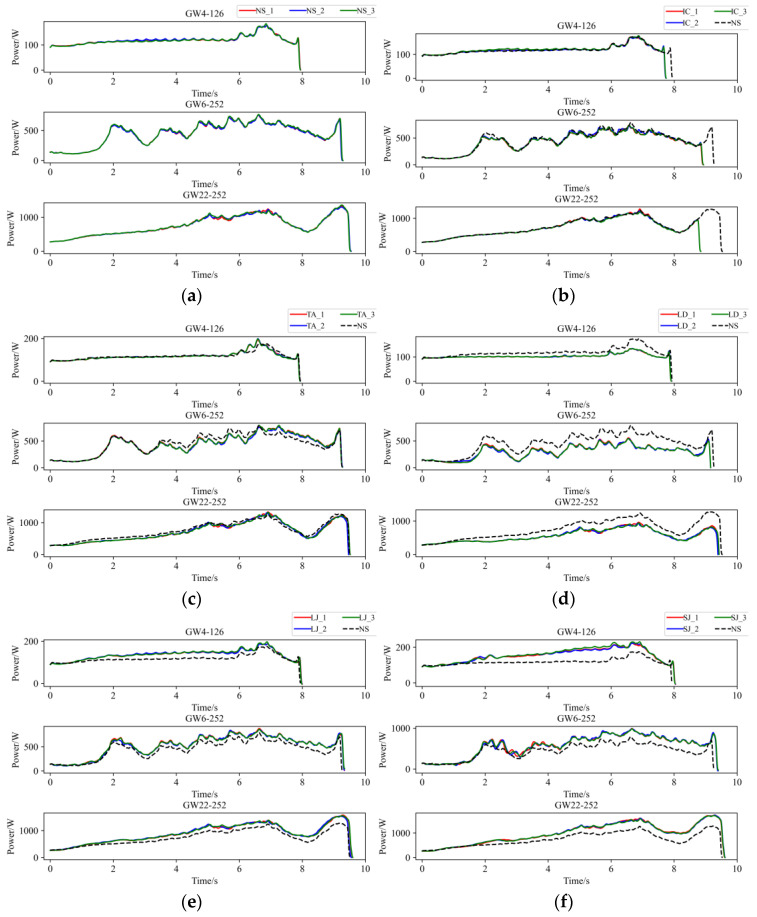
Operation power signal. (**a**) Normal state (NS), (**b**) inadequate closing (IC), (**c**) three-phase asynchrony (TA), (**d**) linkage decoupling (LD), (**e**) light jamming (LJ), (**f**) severe jamming (SJ).

The operational power signals of HVDs under different operating conditions are shown in Figure 5. Nevertheless, given that the start-up phase represents an intrinsic motor characteristic with a very brief duration, it typically lacks significant state information regarding the HVD. Therefore, in this study, the peak at startup is removed. From Figure 5, it can be observed that for signals under the same HVD state, we present the results of three measurements. These results indicate that the operational power signals of the HVD exhibit good repeatability under identical conditions. This is because when the HVD operates under the same mechanical state, the force acting on the rotating spindle of the HVD is relatively stable at the corresponding moment. Only when the mechanical state changes does the torque driving the HVD mechanism vary, resulting in corresponding changes in the motor power. Additionally, the tests revealed that there were no significant differences in the operational signals of the HVD when collected at higher sampling frequencies and bandwidths. Consequently, the subsequent experiments in this study maintained a sampling rate of 4000 Hz. The analysis process for opening and closing operations is similar; however, the closing operation contains more critical information about certain conditions, such as IC. Therefore, this paper will focus primarily on analyzing the closing process of HVDs.

In the NS, the GW4-126 operates with uniform rotation during the initial phase of the mechanism, resulting in stable force on the main shaft. The motor only outputs power to overcome friction, whereas in the later stage, due to the engagement of the contact fingers, the power signal exhibits peaks, with a maximum value of 176 W. Meanwhile, the motors of GW6-252 and GW22-252 overcome the weight of the mechanism during the early operation stage, leading to noticeable fluctuations and an upward trend in power; peaks also occur at the moment of contact engagement. The GW6-252 reaches a maximum power of 747 W during the ascent phase and the GW22-252 peaks at 1289 W during the final contact engagement stage. The average power of GW4-126 is 121 W, GW6-252 is 454 W, and GW22-252 is 745 W. There are significant differences in the action times, maximum and average power, as well as their respective timestamps among the three HVDs. In the IC state, the force conditions affecting the HVD mechanism are largely similar to those in the NS, except that the limit switch is triggered prematurely in the final operation stage. Thus, the signals of three HVDs all closely mirror that of the NS, but with the exception of a segment missing towards the end of the signal. In the TA state of the GW4-126, when the contacts are fitting, the internal contact fingers of phases B and C make contact with the terminals prematurely. This results in the signal initially exceeding and then falling below the NS signal and a slight increase at maximum power. However, the signals of GW6-252 and GW22-252 display slightly different behavior, with the signal first falling below and then exceeding the NS signal. The maximum value of GW6-252 remains nearly unchanged. In contrast, the maximum power of GW22-252 shows a slight increase, but the time at which this maximum occurs has shifted. This difference arises because, during the simulating process, the travel of the phase A mechanism was slightly adjusted to lag behind. Despite this variation, the overall characteristics of both are relatively similar. In the LD state, due to the motors of three HVDs exclusively actuating only one phase mechanism, the force on the main shaft is reduced compared to the NS throughout the operation, so there is a noticeable reduction in the amplitude of the power signal throughout the operation process when compared to the NS. In the case of LJ, the additional tension from the elastic belt increases the torque acting on the HVD mechanism, then the increased force on the HVD mechanism leads to a rise in motor torque, resulting in the overall amplitude of the power signal being higher than under NS. The maximum power of GW4-126 increased to 190 W, while GW6-252 saw an increase to 947 W, and GW22-252 increased to 1583 W. In the SJ state, the impact of the fault on the HVD is consistent with that observed in the LJ state, but this characteristic becomes more pronounced. The maximum power of GW4-126 increased to 219 W, GW6-252 to 997 W, and GW22-252 to 1687 W.

**Figure 6:**
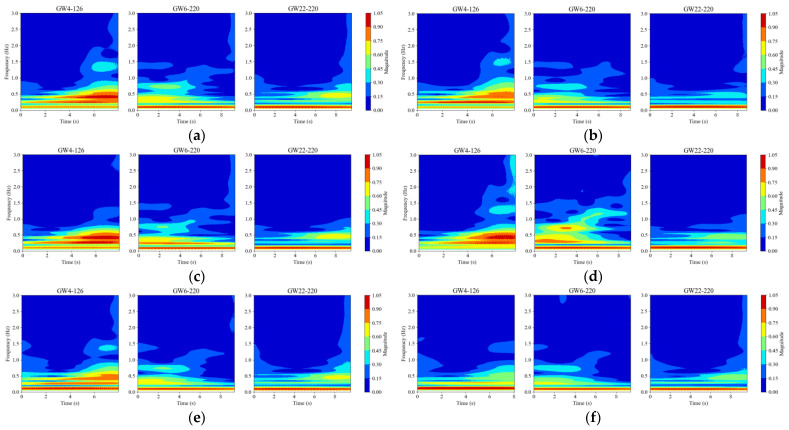
The results after wavelet transform. (**a**) Normal state, (**b**) inadequate closing, (**c**) three-phase asynchrony, (**d**) linkage decoupling, (**e**) light jamming, (**f**) severe jamming.

Continuous Wavelet Transform (CWT) is a powerful time-frequency analysis tool, particularly effective in handling non-stationary signals. To further explore the potential time-frequency commonalities in the operational signals of the three HVDs, this study applies CWT for signal analysis. Considering that the operational power signals of the HVDs are transient signals and that the Morlet wavelet exhibits excellent time-frequency localization capabilities, making it especially suitable for processing transient signals, then Morlet wavelet is selected as the wavelet basis. As shown in Figure 5, the motor power signals of the HVDs contain significant DC components (bias), and there are substantial differences in the overall power amplitudes of the operational signals among the different HVDs. Therefore, before applying CWT, the signals were normalized, and the DC components were filtered out using Fast Fourier Transform (FFT) to prevent the DC component from disproportionately affecting the time-frequency distribution of the signal energy. The CWT results for each operational signal are illustrated in Figure 6.

From Figure 6, it can be observed that during operation, the signal frequencies of the three HVDs are primarily concentrated in the ultra-low frequency range (0–1.5 Hz). The frequency distributions of the operational signals for the same HVD under different states are relatively similar. All three HVDs exhibit a strong energy distribution around 0–0.2 Hz, but they show significant differences in other time-frequency characteristics. For GW4-126, the operational signals display a strong energy distribution in the latter half of the operation period, particularly in the 0.35–1.0 Hz range. In the IC, TA, LJ, and SJ states, the signals demonstrate a decreasing trend in energy at 1.4 Hz around 7 s, with the time-frequency characteristics of IC and LJ being quite similar, while SJ also shows a decline in energy at 0.5 Hz. The frequency distribution characteristics of GW22-220 are somewhat similar to those of GW4-126, with operational signals also exhibiting strong energy distribution in the 0.35–1.0 Hz range during the latter half of the operation period. However, GW22-220 does not display the same time-frequency characteristics across different states as GW4-126 does. For instance, GW6-252 shows no significant differences between the LJ state and the NS. The operational signals of GW6-252 differ considerably in time-frequency characteristics from the other two HVDs, primarily exhibiting strong energy distribution in the 0.3–0.8 Hz range during the first half of the operation period. Additionally, the trends observed in the fault state of GW6-252 show almost no commonality with the other two HVDs.

The significant structural differences among the three HVDs result in considerable variations in their motor operation power curves, making it difficult to find commonalities based on specific time-domain features, such as the amplitude of the maximum power, the timing of the maximum values, and the average power. Similarly, in terms of time-frequency characteristics, the three HVDs do not exhibit similar frequency distribution patterns. Consequently, it is challenging to derive features that could unify the common influences of different states on the HVDs from either the time domain or the time-frequency perspective. But it can be seen that characteristic differences caused by the same type of fault are remarkably similar. Thus, this explains that the method employed in this paper, which involves aligning the fault signals with normal signals and then calculating the differences to obtain the FDS, is indeed a reasonable approach.

By processing the operational signals under different states and converting them into ARPM two-dimensional plane data, the resulting visualizations are shown in Table 4, The legend enhances the color differentiation, making the visual distinctions more pronounced. To provide better clarification, features have been highlighted in red.

From Table 4, it can be observed that the amplitude variations and relative changes in sequence values in the time-series signals are represented in the relative position matrix through the thickness, density, and color intensity of the stripes. Specifically, for the label “1”, it can be observed that in the IC, the ARPMs of the three HVDs exhibits distinct stripes on the right and bottom, indicating significant abnormalities in the final stages of HVDs operation. For label “2”, the ARPMs of the three HVDs in the TA shows an intersection of bright horizontal stripes and dark vertical stripes, demonstrating fluctuations in output magnitude compared to the NS during operation. For label “3”, the ARPMs of all three HVDs appears darker in the marked area under the LD. In contrast, for label “4”, the ARPMs show a localized brightening, which is opposite to the image characteristics of label “3”. This corresponds to the observation that HVDs exhibit greater output in the LJ state compared to the normal state, while showing reduced output in the LD state. For label “5”, the ARPM of the three HVDs displays a similar characteristic to that of label “4”, but with a more pronounced intensity. Due to differences in action time, mechanical design, and fault simulation levels, the positions and shapes of the stripes in the ARPMs are not completely identical for the three HVDs under the same states. However, overall, the ARPM between different classes exhibits distinct stripe color characteristics, which facilitate CNNs in better feature extraction and learning. Meanwhile, for different HVDs under the same state, the RPM demonstrates a high degree of intra-class similarity, aiding the CNN in adapting to and recognizing samples not included in the training set.

**Figure 7:**
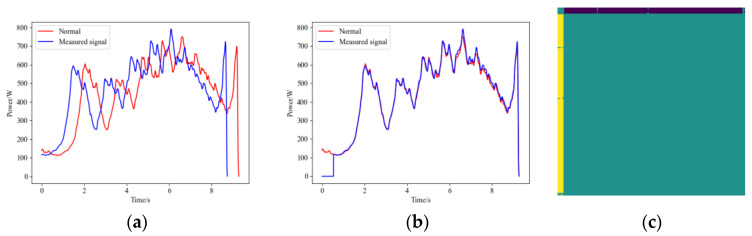
The result of waveform registration. (**a**) The operational signal after the GW22-252 HVD’s travel changing, (**b**) the operational signal after registering, (**c**) the ARPM after travel changing.

After the HVD undergoes maintenance or adjustment, its mechanism travel may change. Specifically, for the open position of the HVD, there may be no reference travel point, so maintenance personnel typically do not fine-tune it to a precise position. When the HVD’s open position travel is advanced following maintenance or adjustment, its operational signal will appear as shown in Figure 7a. In this state, the HVD is still considered to be functioning normally. However, if this signal is not properly aligned with the recorded NS signal, resulting in misalignment, the calculated FDS may contain incorrect information, leading to errors in the diagnostic model. The operational signal after proper alignment is shown in Figure 7b, and the ARPM after the GW22-252 HVD’s travel-changing operational signal is shown in Figure 7c. In light of this situation, we have also included samples with adjusted travel in the dataset.

**Figure 8:**
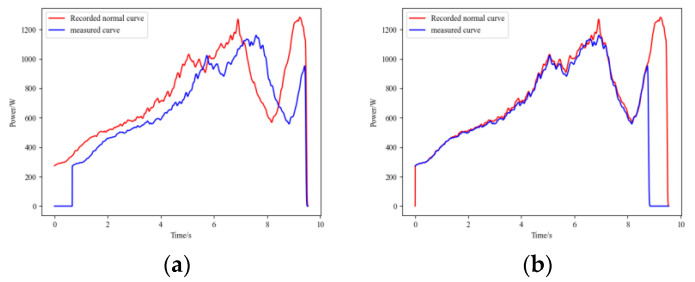
Comparison between algorithms of waveform registration. (**a**) The method before improvement; (**b**) The proposed improved method.

Additionally, if the waveform registration algorithm is unreliable and there are significant errors in curve alignment, the resulting FDS may contain erroneous information. Figure 8 shows the matching results between the IC signals and normal operational signals for GW22-252, both before and after improvement. It can be observed that the method before improvement exhibits a sharp rise in the latter part of the signal curve, resulting in an excessively high cross-correlation coefficient and leading to alignment errors.

To further investigate the impact of noise on the waveform registration algorithm proposed in this paper, different intensities of Gaussian white noise were added to the HVD operation signals to control the signal-to-noise ratio (SNR). We compared the accuracy of the waveform registration algorithm under different SNR conditions with and without filtering. In terms of filter selection, we chose Gaussian filtering because, while low-pass filtering can introduce ringing effects and lose signal fluctuations’ details, Gaussian filtering quickly smooths the signal while preserving waveform details. The comparative results are shown in Table 5.

The data indicate that as the SNR decreases, the accuracy of the algorithm without filtering drops rapidly. In contrast, when signal filtering is applied, the robustness of the proposed waveform registration algorithm is significantly enhanced.

## 4. Result and Analysis

The following sections discuss in detail the data of dataset distribution, and the method we proposed was tested. For the construction of the neural networks, Python 3.11 was employed alongside the PyTorch module (version 2.0.1+cu118), with the computations being facilitated by an RTX 4090 GPU.

### 4.1. Dataset Distribution

Sample data were collected through operations of the HVDs, and the distribution of data within the dataset created for this experiment is detailed in Table 6. Among them, one NS operational signal is taken for each of the three models of HVDs as the recorded NS signal.

### 4.2. Model Tranning and Result Analysis

#### 4.2.1. Model Testing on Single Type of HVD

In this part, the model recognition performance for single type HVDs will be tested first. Among the dataset, 60% of the samples were randomly selected as the training dataset, 20% as the validation dataset, and 20% as the test dataset. To avoid randomness of training, each model was trained five times, with each training sample being re-divided.

To better measure and compare the performance of the models, evaluation metrics including accuracy, precision, recall, and F1-score are introduced for the test. Higher values for these metrics indicate a better diagnostic performance of the model. After training, the average performance of the model on the test set was calculated as the final result. The computations can be carried out using the following formulas:

(12) $$\mathrm{Accuracy}=\frac{\mathrm{TP}+\mathrm{TN}}{\mathrm{TP}+\mathrm{TN}+\mathrm{FP}+\mathrm{FN}}\times 100\%,$$

(13) $$\mathrm{Precision}=\frac{\mathrm{TP}}{\mathrm{TP}+\mathrm{FP}}\times 100\%,$$

(14) $$\mathrm{Recall}=\frac{\mathrm{TP}}{\mathrm{TP}+\mathrm{FN}}\times 100\%,$$

(15) $$F1=2\cdot \frac{\mathrm{Precision}\cdot \mathrm{Recall}}{\mathrm{Precision}+\mathrm{Recall}}\times 100\%.$$

here TP (True Positive) represents the number of instances correctly classified as a specific state type, TN (True Negative) refers to instances correctly classified as not belonging to that state type, FP (False Positive) indicates instances incorrectly classified as a specific state type, and FN (False Negative) refers to instances that belong to a specific state type but were incorrectly classified as another type. All models in this study are designed for multi-class classification tasks, and precision, recall, and F1 score are calculated using class averages.

In the training process, the Adam optimizer is uniformly utilized, with a batch size set at 32. The learning rate is set to 1 × 10^−6^ and the training epochs are all set to 200 epochs. The loss function employed is cross-entropy.

**Figure 9:**
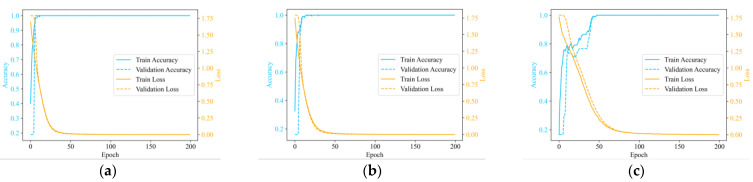
Training process of the single type HVD of the proposed method. (**a**) On GW4-126, (**b**) on GW6-252, (**c**) on GW22-252.

Figure 9 shows the accuracy and loss changes during a particular training session of our proposed method. The loss value decreased rapidly, eventually reaching a convergence state before 200 epochs of training. The validation loss displayed a nearly identical trend, with both values being very close to each other. There were no signs of overfitting or underfitting in the model. The accuracy eventually stabilized at 100%, all states were accurately recognized.

To evaluate the effectiveness of the proposed states diagnosis method, we compare it with the other recent methods, including: SVM, BP, Bi-LSTM, Transformer. SVM is suitable for handling small sample high-dimensional data, while BP networks can effectively manage complex nonlinear relationships. Bi-LSTM excels in time series data, and the Transformer model efficiently captures long-range dependencies and complex features. The input for these models consists of deactivated peak operational signals of the HVD, which have been downsampled and scaled to a length of 224. Additionally, the traditional RPM transformation method is introduced and converts the sample into 224 × 224 two-dimensional images through RPM transformation and finally classifies them using the IAlexNet CNN.

To demonstrate the advantages of using ARPM to convert one-dimensional time series into two-dimensional images for recognition and classification with CNN, we also introduce a comparative model based on the FDS data processing framework. In the classifier, we select representative machine learning model SVM and the deep learning model Transformer, which has efficient learning capabilities for time series data.

The performance of each method is based on the metrics provided in Equations (12)–(15) is shown in Table 7. The calculation of precision, recall, and F1-score begins by determining the values for each class, followed by computing the average across all classes.

From Table 7, we can see that training and testing for a certain type of HVD and its internal samples can easily achieve a recognition accuracy of 100%. This is because samples within the same class of the same HVD type often exhibit high levels of redundancy and similarity, leading to a relatively homogeneous set of features. Consequently, the classifier can easily learn the different class features. Although the testing accuracy of the model on a single type of HVD does not show a specific advantage compared to other comparison methods, it still demonstrates the ability to train and recognize HVDs of that single type.

#### 4.2.2. Model Adaptability Testing

In this part, the training process of the model will only use one type of HVD for training, while in the evaluation phase, samples from the other two types of HVD will be used as tests. The GW4-126 HVD structure is simple and easy to maintain, with a considerable number of units deployed in substations, making fault data more readily available. Therefore, this study focuses on the GW4-126 as the training subject. The testing process is described as follows: First, all samples of the GW4-126 HVD are used as the training set to train the corresponding model and ensure model convergence through learning curves. Next, the GW6-252 dataset is divided into a 1:1 ratio, with one part designated as the validation set and the other part combined with the GW22-252 dataset to form the test set. The validation set is utilized to assess the accuracy of the trained model, allowing for initial evaluation and fine-tuning of the model parameters to achieve the final model. Finally, the validation set is used for performance testing, and evaluation metrics are calculated.

The performance of each method tested on GW6-252 test set and GW22-252 dataset are shown in Table 8. From Table 8, it can be seen that even when trained on data from only one type of HVD, the proposed method demonstrates the best performance in recognizing data from the other two HVD types. The average accuracy and F1-score for GW22-252 and GW6-252 reach 98.27% and 98.25%, respectively. In contrast, traditional methods exhibit weak generalization ability in recognizing data from the other two HVDs, with performance metrics that are notably low and lacking practical application value. Among these, the SVM and Transformer models perform slightly better. Therefore, algorithms incorporating the FDS data processing framework, represented by these two models, show some adaptability in recognition. The average accuracy of FDS-SVM and FDS-Transformer achieves 83.07% and 78.84%, respectively, with average F1-scores of 81.50% and 79.40%. However, there remains a significant gap compared to the method proposed in this study.

**Figure 10:**
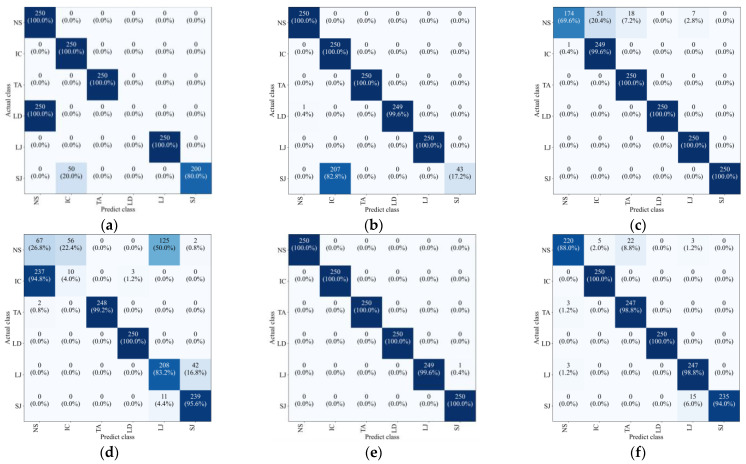
Confusion matrics in adaptability testing of (**a**) FDS-SVM on GW6-252 test set, (**b**) FDS-SVM on GW22-252 dataset, (**c**) FDS-Transformer on GW6-252 test set, (**d**) FDS-Transformer method on GW22-252 dataset, (**e**) the proposed method on GW6-252 test set, (**f**) the proposed method on GW22-252 dataset.

**Figure 11:**
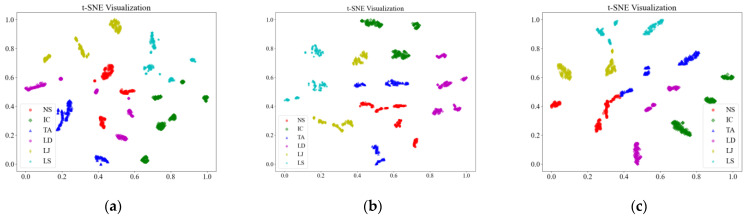
t-SNE comparison of (**a**) FDS-SVM, (**b**) FDS-Transformer, (**c**) the proposed method.

To further enhance the comparison among the three models with adaptive recognition capabilities, the confusion matrics and t-SNE (t-Distributed Stochastic Neighbor Embedding) plots are also introduced. The confusion matrix can be visualized through methods such as heat maps, making the analysis more intuitive and facilitating quick evaluation of the model, and the confusion matrics of three models are shown in Figure 10. t-SNE is a dimensionality reduction technique that can map high-dimensional data to a lower-dimensional space. This facilitates the visualization of data, making it easier for us to understand the distribution and clustering of the data and it. All samples were input into the model, and the outputs were visualized using t-SNE, as shown in Figure 11.

Figure 10 further illustrates the superiority of the proposed method. In the case of GW6-252, there was only one misclassification, while for GW22-252, the misclassifications primarily occurred in the NS and SJ categories. This may be attributed to the lower signal repetitiveness of GW22-252 compared to GW4-126, as well as differences in how each type of HVD simulates the jamming condition. The FDS-Transformer shows poorer sensitivity in detecting the IC state and exhibits similar characteristics to the proposed method in the NS and jamming states, but with a higher number of misclassifications. In contrast, FDS-SVM demonstrates characteristics that are inconsistent with the aforementioned two models, likely due to SVM’s nonlinear decision boundary in the sample space, and it has similar overall accuracy to that of FDS-Transformer. These findings emphasize the high generalization ability of the proposed method. From Figure 11, it is evident that the proposed method exhibits stronger cohesion among samples of the same state compared to the other two models. In contrast, the other two models show some categories with disorganized distributions in the sample space, resulting in poor cohesion for certain class samples. These observations underscore the high generalization ability of the proposed method.

#### 4.2.3. Time Complexity Testing

In the context of HVD state diagnosis, it is essential for the diagnostic algorithm to possess a certain level of real-time capability. Therefore, it is necessary to assess the time complexity of the model. This paper evaluates the models by processing 500 samples and calculating the average execution time of each sample as a performance metric. The result of complexity testing is shown in Table 9.

From Table 9, it can be observed that the proposed method does not excel in terms of time complexity metrics. However, its execution speed is comparable to that of other neural network methods, remaining within a minimal time range. This slight difference is unlikely to have any significant impact in practical applications.

#### 4.2.4. Ablation Study

**Figure 12:**
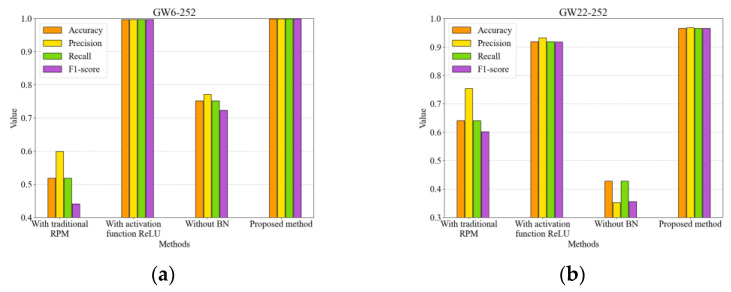
Ablation result. (**a**) On GW6-252 test set, (**b**) on GW22-252 dataset.

The ablation study demonstrates the critical components of the proposed method and their contributions to overall performance. In this study, three control groups were established for the ablation experiment: replacing ARPM with the traditional RPM, substituting the activation function with ReLU, and removing BN. The testing is still based on the adaptive capability testing process, and the performance of each model is shown in Figure 12. We observed that, apart from the model that replaced the activation function, which showed metrics slightly lower than those of the proposed method on the GW6-252 dataset, the generalization performance of the other models significantly declined. This underscores the importance of each component in the FDS-ARPM-CNN model.

#### 4.2.5. Expansion Study

**Figure 13:**
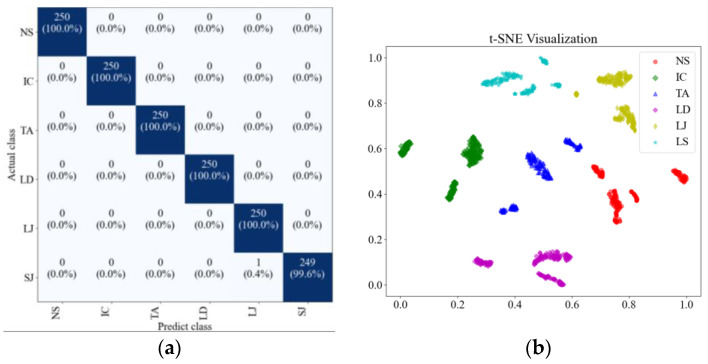
The result of expansion study. (**a**) confusion matrix on GW22-252 dataset, (**b**) t-SNE.

To further validate the potential for performance improvement of the proposed model, we conducted an expansion experiment. In addition to the GW4-126 dataset, we incorporated the validation set of GW6-252, which constitutes 50% of GW6-252 dataset, into the training data. The model was then trained and tested on the GW22-252 dataset. The model performance metrics are presented in Table 10, while the confusion matrix and t-SNE plot are shown in Figure 13. The results clearly indicate that compared to the model trained solely on GW4-126, the model demonstrates improved recognition performance on the GW22-252 dataset. During five consecutive tests, only one instance of SJ was incorrectly identified as LJ. In Figure 13b, intra-class cohesion has strengthened, while inter-class dispersion has become more distinct.

## 5. Conclusions

To achieve intelligent state diagnosis of HVDs in substations while minimizing complexity and cost and addressing the challenges of limited fault samples for specific HVD types that hinder machine learning applications, this paper proposes a novel diagnostic method with adaptive recognition capability. This method trains a neural network model using the operational power signal from one model of HVD to diagnose states on data from other HVD models, achieving high recognition accuracy.

Specifically, the method computes the difference between the measured operational signals and the recorded normal operational curves of HVDs to obtain a new fault difference signal. This signal is then converted into an ARPM matrix to enhance underlying features, which is subsequently classified using an improved CNN.

Experimental results demonstrate that the proposed method achieved 100% accuracy in identifying the status of a specific type of HVD on the experimental test set, trained using its own training dataset. In the adaptive testing, where the model is trained solely on GW4-126 HVD data and used to recognize the states of GW6-252 and GW22-252 HVDs, traditional machine learning and deep learning approaches show poor adaptive classification capability. In contrast, the proposed method achieves an average recognition accuracy and F1-score exceeding 98%. Additionally, the performance of the time-series models based on the FDS introduced in this paper is significantly inferior to that of the proposed method. Ablation studies further confirm the effectiveness of the targeted optimization measures in enhancing model performance. Moreover, through extended experiments, we found that increasing the variety of HVD types in the training data also improves the recognition accuracy of the untrained HVD types. Although the proposed model algorithm does not excel in terms of time complexity, its improvement in identifying untrained HVD states is more significant for applications compared to its slight differences with other algorithms.

Notably, this paper introduces a one-to-many feature data collection method for HVDs, which involves installing sensors at the junction of the HVD’s power supply branch. This approach enables recognition and monitoring of HVD states with minimal workload and equipment costs, ensuring significant feasibility for real-world applications.

However, this study tests only six distinct HVD states and three types. In practice, various complex operational states of HVDs and specialized types, such as disconnectors within GIS, exist. Therefore, further exploration of these aspects is necessary in future research.

## Figures and Tables

**Table 1:** The structure of the proposed deep learning network.

Type of Layers	Shape of Output	Parameters
Input layer	(3, 224, 224)	-
Convolutional layer 1	(96, 55, 55)	Kernel size: 11 × 11 × 96, stride:4, padding:2
Batch Normalization (BN) layer 1	(96, 55, 55)	With activation function: GELU
Maxpooling layer 1	(96, 27, 27)	Kernel size: 3 × 3, stride:2, padding: 0
Convolutional layer 2	(256, 27, 27)	Kernel size: 5 × 5 × 256, stride:1, padding:2
BN layer 2	(256, 27, 27)	With activation function: GELU
Maxpooling layer 2	(256, 13, 13)	Kernel size: 3 × 3, stride:2, padding: 0
Convolutional layer 3	(384, 13, 13)	Kernel size: 3 × 3 × 384, stride:1, padding:1
BN layer 3	(384, 13, 13)	With activation function: GELU
Convolutional layer 4	(256, 13, 13)	Kernel size: 3 × 3 × 256, stride:1, padding:1
BN layer 4	(256, 13, 13)	With activation function: GELU
Convolutional layer 5	(256, 13, 13)	Kernel size: 3 × 3 × 256, stride:1, padding:1
BN layer 5	(256, 13, 13)	With activation function: GELU
Maxpooling layer 3	(256, 6, 6)	Kernel size: 3 × 3, stride:2, padding: 0
Flatten	9216	-
Dense net 1	4096	Dropout: 0.5, activation function: GELU
Dense net 2	4096	Dropout: 0.5, activation function: GELU
Output layer	6	activation function: Softmax

**Table 2:** The parameters of the signal acquisition device.

Parameters		
Overall Accuracy	Sampling Rate (Max)	Bandwidth (−3 dB)
±0.5%	20 ksps	15 kHz

**Table 3:** High-Voltage Disconnector (HVD)states simulation scheme.

Types of States	The Cause of State	Simulation Methods
Inadequate Closing (IC)	The intricate components, like bolts and gears within the limit switch, are susceptible to deformation due to prolonged outdoor exposure.	The bolt on the limit block is lengthened, so that the time of hitting the limit block and the stop action time is advanced.
Three-phase Asynchrony (TA)	Improper debugging by on-site installation, debugging, or maintenance personnel, or deformation of connecting rods.	Adjusting the connecting rod between drive phase and driven phases in the open state, thereby causing one phase lagging behind the other two phases.
Linkage Decoupling (LD)	Loosening or detachment of components within the three-phase linkage mechanism.	the bolt nut that secures the connecting rod between drive phase and driven phases was deliberately loosened, and the connecting rod was disengaged. Consequently, the structures of two driven phase could not be simultaneously actuated by the motor.
Light Jamming (LJ)	Oxidation corrosion and the depletion of lubricating oil among moving components.	Employing multiple elastic bands to link the bases of the supporting insulators on the HVD.
Severe Jamming (SJ)		Increasing the number of elastic bands on the basis of LJ.

**Table 4:** Conversion of operational signals in Adjusted Relative Position Matrix (APRM).

Types of HVDs	GW4-126	GW6-252	GW22-252
Normal State (NS)	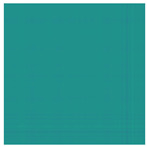	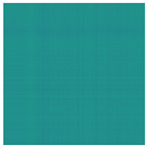	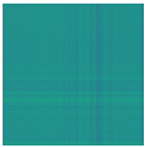
IC	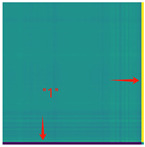	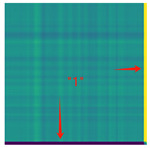	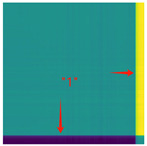
TA	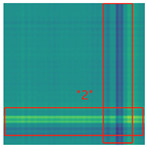	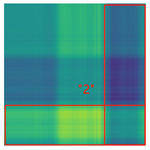	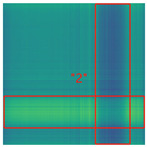
LD	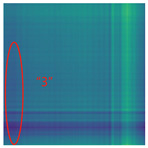	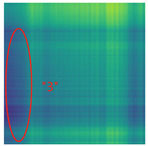	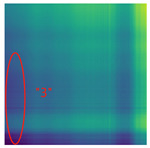
LJ	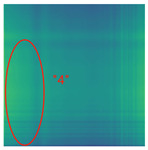	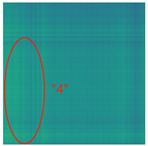	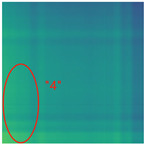
SJ	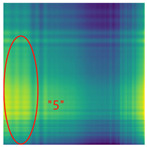	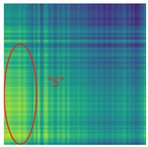	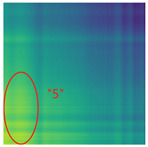

**Table 5:** The comparison of waveform registration algorithms before and after introducing filtering.

Method	Accuracy			
	60 db	55 db	50 db	45 db
Without filtering	100%	93.4%	81.4%	45.2%
With filtering	100%	100%	100%	100%

**Table 6:** Dataset distribution.

HVD Type	Types of States					
	NS	IC	TA	LD	LJ	SJ
GW4-126	100	100	100	100	100	100
GW22-252	100	100	100	100	100	100
GW6-252	50	50	50	50	50	50

**Table 7:** Performance comparison of various methods in single type of HVDs testing.

Evaluation Metrics (The Average of Three HVD)	SVM	BP	Bi-LSTM	Transformer	RPM-CNN	FDS-SVM	FDS-Transformer	FDS-ARPM-CNN
Accuracy	1.0	1.0	1.0	1.0	1.0	1.0	1.0	1.0
Precision	1.0	1.0	1.0	1.0	1.0	1.0	1.0	1.0
Recall	1.0	1.0	1.0	1.0	1.0	1.0	1.0	1.0
F1-score	1.0	1.0	1.0	1.0	1.0	1.0	1.0	1.0

**Table 8:** Performance comparison of various methods in adaptability testing.

Methods	Types of HVDs	Accuracy	Precision	Recall	F1-Score	Average Accuracy	Average F1-Score
SVM	GW6-252	55.03%	61.22%	55.03%	49.76%	44.18%	37.38%
	GW22-252	33.33%	20.24%	33.33%	25.00%		
BP	GW6-252	50.13%	38.87%	50.13%	42.47%	43.40%	35.75
	GW22-252	36.66%	25.59%	36.66%	29.03%		
Bi-LSTM	GW6-252	49.60%	40.00%	49.6%	39.78%	31.87%	27.08%
	GW22-252	14.13%	16.84%	14.13%	14.38%		
Transformer	GW6-252	59.60%	50.86%	59.60%	51.56%	57.37%	50.76%
	GW22-252	55.13%	52.69%	55.13%	49.99%		
RPM-CNN	GW6-252	16.67%	2.78%	16.67%	4.76%	18.70%	7.26%
	GW22-252	20.73%	9.49%	20.73%	9.75%		
FDS-SVM	GW6-252	80.00%	72.22%	80.00%	74.41%	83.07%	78.84%
	GW22-252	86.13%	92.38%	86.13%	83.27%		
FDS-Transformer	GW6-252	94.87%	96.04%	94.87%	94.50%	81.50%	79.40%
	GW22-252	68.13%	61.72%	68.13%	64.30%		
FDS-ARPM-CNN	GW6-252	99.93%	99.93%	99.93%	99.93%	98.27%	98.25%
	GW22-252	96.60%	96.81%	96.60%	96.57%		

**Table 9:** Model Complexity of different methods.

Evaluation Index	SVM	BP	Bi-LSTM	Transformer	RPM-CNN	FDS-SVM	FDS-Transformer	FDS-ARPM-CNN
Time Complexity (ms)	3 × 10^−3^	1.07	0.61	2.71	1.05	1.12	4.25	2.10

**Table 10:** Performance of the proposed method in expansion study.

Methods	Types of HVDs	Accuracy	Precision	Recall	F1-Score	Average Accuracy	Average F1-Score
FDS-ARPM-CNN	GW4-126	100%	100%	100%	100%	99.98%	99.98%
	GW6-252	100%	100%	100%	100%		
	GW22-252	99.93%	99.93%	99.93%	99.93%		

## Data Availability

The data presented in this study are available on request from the corresponding author.
